# Uncommon Presentation of Hidrocystoma in a 14-Year-Old Girl

**DOI:** 10.3390/diagnostics16121772

**Published:** 2026-06-09

**Authors:** Agata Maria Kawalec-Rutkowska

**Affiliations:** Department of Anatomy, Institute of Medical Sciences, University of Opole, Oleska St. 48, 45-052 Opole, Poland; agata.kawalec@uni.opole.pl

**Keywords:** hidrocystoma, retroauricular tumor, child, ultrasonography, earrings

## Abstract

Hidrocystoma is a rare benign cystic tumor of sweat gland origin, most commonly located in the periorbital region, with uncommon occurrence in the retroauricular area. This article presents a case of a 14-year-old patient with a retroauricular mass present for approximately four years, which had remained stable in size until a gradual enlargement was observed over the preceding 11 months. The lesion was associated with intermittent fluid discharge and periodic episodes of local skin inflammation, likely related to mechanical irritation from earrings worn by the patient. Otherwise, the lesion was asymptomatic, with no persistent pain or systemic signs of infection. Clinical examination revealed a well-circumscribed, cystic lesion located in the retroauricular region. Ultrasonographic evaluation demonstrated features consistent with a benign cystic structure. Based on clinical and imaging findings, the lesion was qualified for surgical excision. Complete removal of the mass was performed without complications. Histopathological examination confirmed the diagnosis of hidrocystoma. The postoperative course was uneventful, with no recurrence observed during follow-up. This case highlights a rare location and atypical clinical course of hidrocystoma in an adolescent patient, emphasizing the role of clinical assessment and ultrasonography in preoperative evaluation, as well as the potential impact of chronic mechanical irritation on local inflammatory episodes. Surgical excision remains an effective definitive treatment.

**Figure 1:**
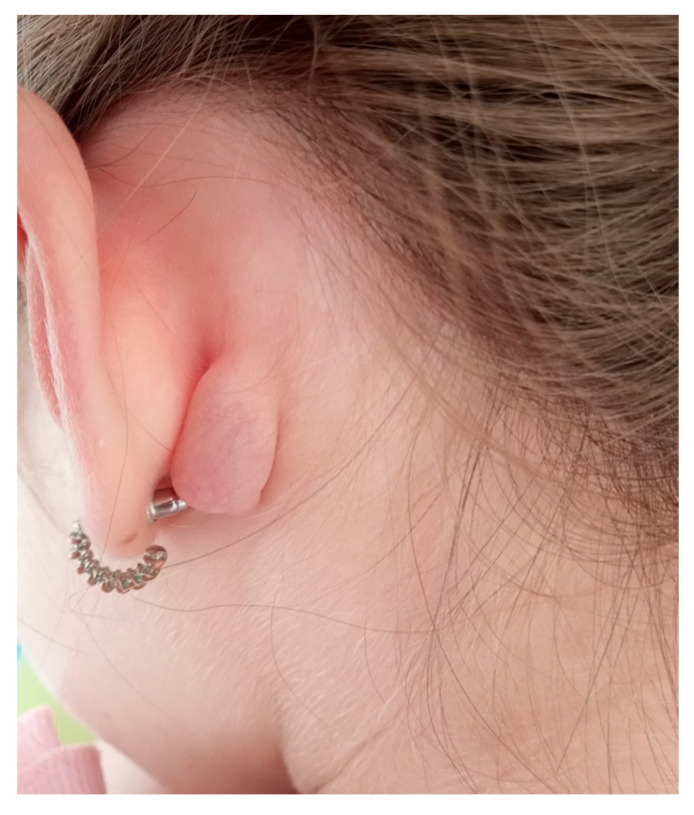
Clinical presentation of the 14-year-old patient showing a soft, non-tender mass located behind the left auricle. The overlying skin appeared normal, without signs of inflammation or discharge, and regional lymph nodes were not enlarged. According to the patient, the retroauricular mass had been present for approximately four years and remained stable in size until a gradual enlargement was observed during the preceding 11 months. The lesion was associated with intermittent clear fluid discharge and periodic episodes of local skin inflammation, likely related to mechanical irritation from earrings worn by the patient. Otherwise, the lesion was asymptomatic, with no persistent pain or systemic signs of infection. Laboratory tests, including complete blood count with differential and C-reactive protein (CRP), were within normal limits.

**Figure 2:**
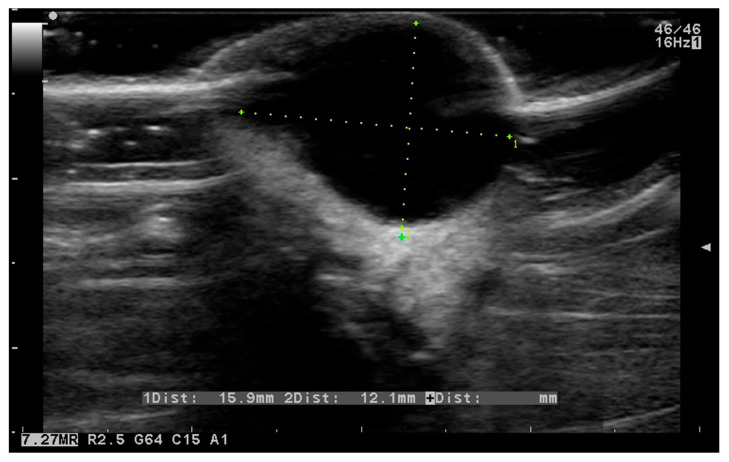
Longitudinal ultrasound view of the retroauricular lesion, demonstrating a well-circumscribed, anechoic, compressible cystic structure. Aloka prosound alpha 6, linear probe. Green caliper lines indicate the ultrasound measurement of the lesion.

**Figure 3:**
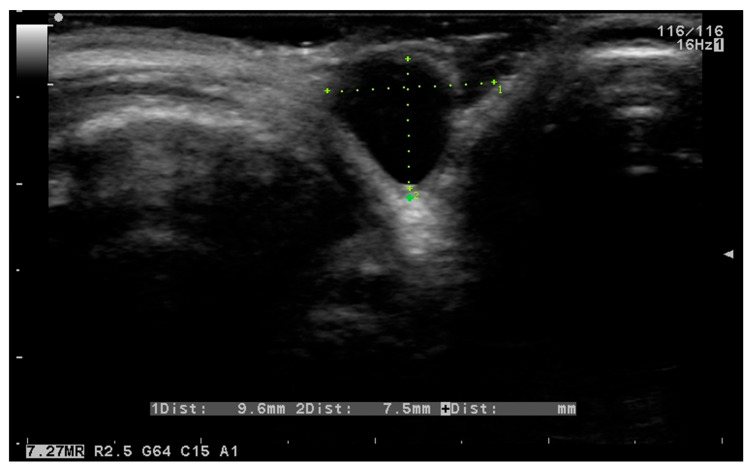
Transverse ultrasound view of the lesion, showing clear boundaries. Aloka prosound alpha 6, linear probe. Green caliper lines indicate the ultrasound measurement of the lesion. Ultrasound examination performed with a linear probe revealed a well-circumscribed, anechoic lesion, compressible with the transducer, with no evidence of increased vascularity on color Doppler imaging. The approximate dimensions of the lesion were [15.9 mm × 9.6 mm × 7.5 mm] (Figure 2 and Figure 3). On ultrasonography, hidrocystomas usually appear as well-circumscribed, anechoic or hypoechoic cystic lesions with posterior acoustic enhancement and absence of internal vascularization on Doppler imaging. In the present case, the lesion was compressible, well-demarcated, and showed no Doppler flow, supporting its benign cystic nature. Unlike epidermal inclusion cysts, which frequently demonstrate internal echogenic debris, keratinous material, or heterogeneous echotexture, hidrocystomas are more often purely cystic and uniformly anechoic. Epidermal inclusion cysts may also show a characteristic punctum and are typically less compressible on ultrasound examination. In addition, laminated keratin debris may appear on ultrasound as mixed hypoechoic or hyperechoic internal echoes. Sebaceous cysts usually present with more heterogeneous internal content and variable vascularity related to inflammation. Nevertheless, ultrasonographic findings are not entirely specific, and histopathological examination remains essential for definitive diagnosis [1,2]. The patient qualified for surgical treatment involving excision of the lesion with subsequent histopathological examination under local anesthesia with 1% lidocaine. Following standard preparation, an incision was made over the lesion. A cystic structure containing clear fluid was identified, carefully dissected from the surrounding tissues, and completely excised macroscopically. Hemostasis was achieved, and the redundant skin was trimmed. The wound was closed with subcutaneous and intracutaneous sutures. Histopathological examination confirmed the diagnosis of apocrine hidrocystoma. Sutures were removed, and the postoperative course was uneventful, with no evidence of recurrence during one year of follow-up. Hidrocystomas are benign cystic lesions arising from sweat glands, showing either apocrine or eccrine differentiation [3]. They are most commonly found on the head and neck, particularly in the periorbital region [4]. Rarer presentations include lesions on the nose [5] and within the auditory canal [6,7]. Lesions occurring outside the head and neck region have also been described, including cases mimicking breast lesions in children and involvement of the genital area [3,8]. The retroauricular localization described in this case is rarely reported in the literature [9,10]. To the best of current knowledge, only isolated cases in adult women have been reported, with no pediatric cases documented [9,10]. This highlights the importance of considering hidrocystoma in the differential diagnosis of retroauricular cystic lesions. The differential diagnosis of cystic retroauricular lesion includes sebaceous cysts, hidrocystomas, and epidermal inclusion cysts [9]. Hidrocystomas typically present as firm, smooth, bluish papules or nodules, with clinical features varying depending on location. The cystic space of an eccrine hidrocystoma contains clear or brown fluid; in the presented case, it was clear. Multiple hidrocystomas can be associated with focal dermal hypoplasia (Jessner–Cole or Goltz–Gorlin syndrome), but the reported patient had a solitary lesion [6]. Surgical excision remains the gold standard for both definitive diagnosis and treatment, ensuring excellent outcomes with a low risk of recurrence, while alternative therapies may be considered for multiple or surgically challenging lesions [6,9].

## Data Availability

The original contributions presented in this study are included in the article material. Further inquiries can be directed to the author.
